# Rheological characterization and fouling deposition behavior of coconut cream emulsion at heat processing temperature range

**DOI:** 10.1002/fsn3.2977

**Published:** 2022-07-27

**Authors:** Avan Maghazechi, Abdorreza Mohammadi Nafchi, Thuan‐Chew Tan, Azhar Mat Easa

**Affiliations:** ^1^ Food Technology Division, School of Industrial Technology Universiti Sains Malaysia Penang Malaysia; ^2^ Food Science and Quality Control Department, College of Agricultural Engineering Science University of Sulaimani Sulaimani Iraq; ^3^ Food Biopolymer Research Group, Food Science and Technology Department, Damghan Branch Islamic Azad University Damghan Iran; ^4^ Renewable Biomass Transformation Cluster, School of Industrial Technology Universiti Sains Malaysia Penang Malaysia

**Keywords:** coconut cream emulsion, heat treatment, protein denaturation, rheological properties

## Abstract

Fouling deposition in the coconut cream emulsion (CCE) is considered a severe technical issue in the industry. Since the fouling deposition results from the heating effect on the CCE bulk, the heat‐induced structural changes in the CCE bulk at different temperatures were rheologically investigated in the first part of this study. The second part applied different heat treatment conditions to investigate generated fouling deposition (GFD). Chemical composition, FTIR, and SEM imaging were used to explore GFDs thoroughly. The increase in viscosity and storage modulus (G′) reflect such heat‐induced changes over the experimental conditions. More structural changes were predicted at around ≥85°C, accompanied by a sharp increase in viscosity and (G′), which was associated with the gelation of CCE. The conformational transition, fat agglomeration in CCE bulk, generated fouling deposits (GFDs) were significant around 70°C. The chemical composition of the GFD has shown an increasing trend in the protein, carbohydrates, and ash, meanwhile fluctuation in the fat contents with increasing temperature. The FTIR peaks showed novel peaks around temperature ≥85°C, which implied new amide groups or new protein conformation. The SEM images provided the different microstructures of GFDs at high‐temperature levels. More likely the GFDs appeared at temperature ≥85°C are a gel deposit layer. These findings strongly suggest that emulsion gelation was the primary cause of coconut cream fouling.

## Graphical abstract

- The heat‐induced changes in coconut cream emulsion (CCE) have been investigated for fouling issue study.
- The conformational transition, fat agglomeration, and flocculation in CCE end up with generated fouling depositions.
- The findings strongly suggest that emulsion gelation was the primary cause of coconut cream fouling.

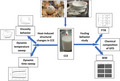

## 1 INTRODUCTION

Coconut cream emulsion extracted from mature fruits of *Cocos nucifera L*. is considered a complex multicomponent fluid, typically composed of fat, protein, carbohydrate, and minerals. Due to this, it is a vibrant medium that can support the growth of all common spoilage microorganisms (Rajamani et al., 2020). There is a confusion among the literature, industry, and consumers regarding the coconut milk and its cream terms. Based on CODEX STAN240‐2003, coconut milk is extracted from grated mature coconut meat (Kernel) by pressing or squeezing with the addition of water as a diluted emulsion that contains not less than 12.7% of total solids, 2.7% of non‐fat solids, and 10% fat. While coconut cream is a pure, undiluted emulsion made from the same mature coconut kernel, it contain a minimum of 25.4% of total solids, 5.4% non‐fat solids, and 20% fat.

Heat treatments such as (cooking, pasteurization, and sterilization) are effective ways to extend the shelf life of coconut milk and its cream (Wattanapahu et al., 2012). Pasteurizing milk at 72°C for 20 minutes provides the necessary short‐term storage by destroying pathogenic microorganisms and degrading enzymes and ultimately improving shelf life and product quality (Deak, 2013). To produce convenient foods and prolong shelf stability and shelf life, the most common commercial coconut milk products use ultrahigh‐temperature (UHT) sterilization (Jirapakkul et al., 2018). The main problem in the production of canned coconut milk is its instability during thermal processing and prolonged holding times. Coconut milk coagulates readily upon heating to 80°C due to the denaturation of heat‐labile proteins. This may produce unacceptable curdled products, particularly in the more concentrated forms of milk (Hagenmaier, 1980; Seow & Goh, 1994). Such coconut milk denaturation problem is followed by increased viscosity due to emulsion flocculation via a bridging mechanism (Tangsuphoom & Coupland, 2005). This coincides with the accumulation of fat globules in the viscous coconut milk bulk, which tends to contain fewer single fat globules to withstand the flow operation (Konkamdee & Saikhwan, 2015). The increased viscosity of coconut milk adversely affects heat transfer during the processing of canned products in static retorts. A longer time was needed to achieve the desired lethal effect in concentrated milk than in a diluted one; consequently, this may result in undesirable chemical changes such as nonenzymic browning (Seow & Gwee, 1997).

On the other hand, it was reported by Pichitvittayakarn et al. that at temperatures higher than 70°C, some components might lose their rheological properties and denature to form a deposit on process heating surfaces (Pichitvittayakarn et al., 2006). The deposit formation is called coconut milk fouling. Fouling is commonly characterized as the accumulation and deposition of unwanted materials on the processing equipment surfaces (Saikhwan et al., 2016). The fouling deposit formation on heat‐exchanger surfaces is still a severe issue in coconut thermal (Law et al., 2009). The study of Pichitvittayakarn et al. (2006) also pointed out that coconut milk fouling has not been extensively researched in comparison to cow milk fouling. It also referred to the importance of further analysis to validate subsequent deposit levels due to thermal processing, which can contribute to an understanding of the relationship among protein, fat, and mineral adsorption on metal surfaces.

Konkamdee et al. also mentioned that there was little work done on coconut milk fouling and that the practiced cleaning protocols used in the coconut milk industry followed the general, broad dairy cleaning protocols (Konkamdee & Saikhwan, 2015). Furthermore, Narataruksa et al. (2010) recommend more research to show that the mineral forming process is entirely separate from the mechanism of particle aggregation through denatured proteins and fat trapping.

The simulation of the mechanisms that generate fouling deposits has aroused considerable industrial interest (Benning et al., 2003). The studies done in the field of dairy fouling show that the fouling rate has been explored extensively to confirm whether it is determined by reactions in the bulk fluid or at the heating surface (Simmons et al., 2007). Belmar‐Beiny et al. presented two main models based on surface and bulk reactions, demonstrating that the latter may have been the controlling factor (Belmar‐Beiny et al., 1993). These bulk reactions lead to the formation of aggregates that are subsequently deposited onto the surface (Dannenberg & Kessler, 1988). Therefore, each fluid fouling reaction relies on chemistry and reaction rates that generate bulk fluid aggregates (Christian et al., 2002).

The fouling issue is more pronounced in coconut cream than in its milk due to the high total solid content in the former. Most research works have focused on coconut milk rather than its cream. During the thermal processing of coconut cream, it is frequent to encounter fouling deposits, which reduces heat transfer efficiency. Manufacturers need to use many cleaning chemicals that are not environment friendly and the extra costs of materials and man labor. Understanding the nature of the foulants deposited on heat surfaces is crucial in developing the knowledge related to fouling hypotheses and mechanisms, improving fouling mitigation, and better process design.

Since the fouling deposition is a result of the heating effect on the CCE bulk, it is preferable to aim to study the changes that occur in the CCE bulk due to the heating, which leads to fouling deposition, before moving on to studying the fouling deposition phenomenon, which may help to find a way to reduce this issue. Therefore, this research work is divided into two parts; (1) an analysis of heat‐induced structural changes in CCE at (40–90°C) temperature range, using rheological properties modeling and visual inspection methods. (2) An investigation of GFDs using the chemical composition results, FTIR peaks, and SEM image under the effect of heat treatments done at selective temperatures (over 2 h of holding time).

## 2 MATERIALS AND METHODS

### 2.1 Coconut cream emulsion preparation

Mature coconuts (11–13 months old) were obtained from local markets in Gelugor (Penang, Malaysia). Mature coconuts have a dry and brown husk, a minimal amount of water in the cavity of the coconut, thick edible endosperm (meat) around 11 mm, and a thin brown layer seed coat (Testa).

Coconuts were deshelled using a conventional coconut cutter, peeled, and then the fresh coconut meat was washed before final shredding. The CCE was obtained when the shredding coconut meat was pressed using a hydraulic presser (without water), followed by a cheesecloth filter to remove fibers. All prepared CCE samples are used freshly without being stored.

### 2.2 Rheological properties analysis

The rheological properties of CCE were examined using Rheology Advantage Instrument Control AR (T.A. Instruments, New Castle, USA). Obtained data were analyzed using Rheology Advantage Data Analysis software V5.4.8 (T.A. Instruments, New Castle, USA). A parallel plate sensor (diameter 40 mm, gap of 100 μm) has been used throughout the tests. In addition, a solvent trap was used to prevent the evaporation of water from the solution. All the experiments were performed in triplicate.

The temperature in this work was chosen from preliminary work, ranging from 10 to 95°C. The current study will investigate the temperature ranges 40–90°C related to heat processing.

#### 2.2.1 Amplitude sweep test

A stress sweep was first conducted to determine CCE's linear viscoelastic region (LVR) as a setup parameter. The tests were carried out at a temperature of 25°C using an oscillation stress range of 0.01–10 Pa at 1 Hz. The storage modulus (G′) versus oscillatory stress plot determined the linear viscoelastic region (Motamedzadegan et al., 2020). As a result, the LVR parameter of CCE was found in the range 0.1–0.4 Pa.

#### 2.2.2 Linear flow ramp

Measurements were conducted at selective temperatures of 40, 60, 70, 80, 85, and 90°C, with a shear rate range 0–500 s^−1^. The power law model was used for fitting the flow curves, $\tau =K\;{\left(\dot{\gamma }\right)}^{n}$, where *τ* is the shear stress (Pa), $\dot{\gamma }$ is the strain rate (s^−1^), *K* is the consistency coefficient in (Pa.s^n^) which is a measure of viscosity, and *n* is the flow behavior index (dimensionless) (Maghazechi et al., 2021).

#### 2.2.3 Dynamic temperature sweep

Two step‐wise temperature ramps were done as follows: (1) heating from 20 to 95°C for 30 min, with a ramp rate of 3°C/min, at 1 Hz and 0.5% shear strain; then (2) cooling from 95 to 20°C for 30 min in the same ramp rate with some modifications (Mezger, 2020).

#### 2.2.4 Dynamic time sweep

The storage modulus (G′) was recorded within 1 h at selective temperatures (40–90°C). The parameters used for this test were as follows: 0.5% strain (within the predetermined LVR of CCE) and 1 Hz frequency (Mezger, 2020).

#### 2.2.5 Dynamic frequency sweep

This test was conducted only for CCE samples after the heat treatment of 90°C/2 h. Under the predetermined LVR, at 0.01–10 Hz, the oscillatory frequency sweep test was carried out at logarithmic progression and constant strain (Mezger, 2020).

### 2.3 Visual inspection

This was conducted in two parts: (1) a visual assessment of CCE structure changes post the selective heat treatments and (2) a manual measurement of CCE layers instability.

#### 2.3.1 Visual assessment

**FIGURE 1:**
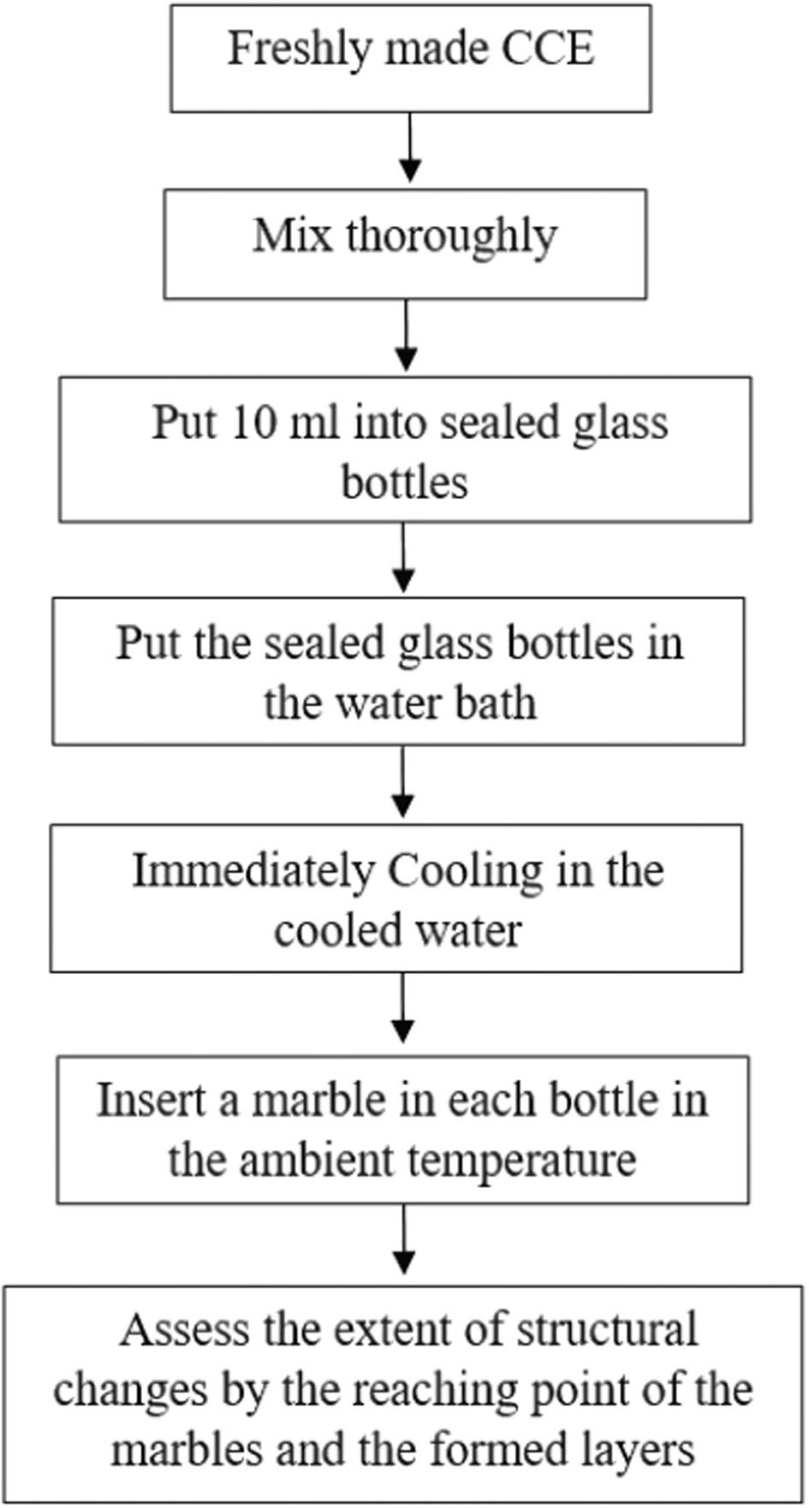
Visual assessment diagram

Visual assessment is generally used to visualize the changes in the structure. Freshly made CCE samples were put into sealed glass bottles (10 mm diameter, 100 mm height) with minimal headspace to minimize evaporation, as explained in Figure 1. Then, each bottle was put in a water bath over holding times of 0, 1, 10, 30, 60, 90, and 120 min at the temperature range 40–90°C. After the heat treatments, each CCE bottle was cooled immediately with cold water to around 25°C, and marble was put in each bottle to visually assess the extent of structural changes and gelation.

### 2.4 Generated fouling deposit (GFDs) analysis

**FIGURE 2:**
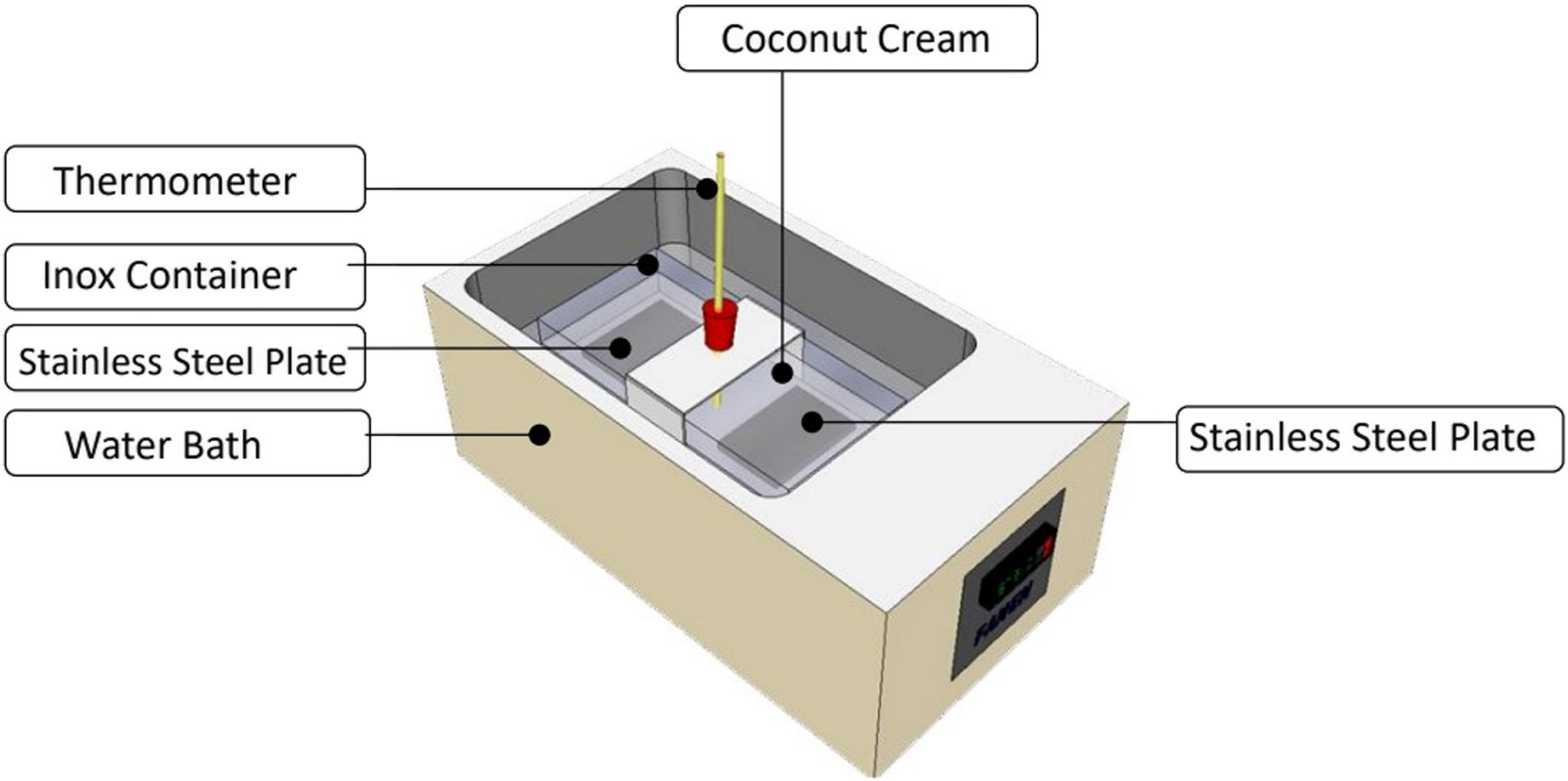
Schematic drawing of the experimental apparatus used for deposit generation after CCE heat treatments

An experimental apparatus at laboratory scale and conditions was conceived to conduct deposition experiments during the heat treatment of CCE. Fouling experiments were done using an 800 ml inox container in a water bath with agitation mode and rectangular dismantled plates put in the bottom of the container (Figure 2). Different heat conditions were used in the range of 40–90°C over 120 min. duration. Each experiment was reproduced at least six times. In FTIR and SEM experiments, all GFDs were washed carefully to remove excess coconut cream, then were put in an air oven at 55–60°C to dry the washing water around the GFDs samples, which may obstruct the electron beam and interfere with imaging clarity. Then, all the GFDs samples were kept in the desiccator before the analysis.

#### 2.4.1 Chemical composition

The proximate composition (total solids, total fat, crude protein [N*5.30], ash, and total carbohydrate) of the raw and CCE deposits were determined according to the procedure described by AOAC (2016). In addition, the mineral elements were determined using the atomic absorption emission spectrophotometer (AAS) model 200A. All analyses were repeated three times.

#### 2.4.2 Fourier infrared spectrometer (FTIR)

The FTIR spectra of GFDs were obtained using Nicolet iS10 Smart OMNI‐transmission FTIR Spectrometer (Thermo Scientific, Madison, WI, USA) as transmission‐mode spectra range 4000–400 cm^−1^ with a resolution of 4 cm^−1^. All the results were analyzed with OMNIC 8.1.11 software (Thermo Scientific, Madison, WI) (Saikhwan et al., 2015).

#### 2.4.3 SEM and macroimaging

The samples were analyzed using Leo Supra 50 V.P. Field Emission SEM equipped with Oxford INCA 400 energy‐dispersive x‐ray microanalysis system (Carl‐Zeiss EVO MA 10, Oberkochem, Germany). The SEM experiments were captured with a magnification of 1000× and 500× and repeated two times. The most significant photographs were presented. CCE deposit samples were not scraped off to avoid their destruction. Test procedures were done according to the detailed description by other researchers with some modifications (Chulibert et al., 2020; Felfoul et al., 2016).

### 2.5 Statistical analysis

Origin Pro 9.0 software (OriginLab Corp., USA) was used to conduct statistical analysis. All rheological experiments, and all GFDs analyses, were replicated three times. Tabular data are presented as arithmetic mean ± standard deviation. Power law model fitting was statistically confirmed using the coefficient of determination (*R* ^2^). Consistency coefficient (*K*) values from power law's fitting model, dynamic time sweep, and proximate analysis were subjected to a one‐way analysis of variance (one‐way ANOVA), followed by Tukey's test for multiple comparisons. A significant level of *p* < .05 was maintained throughout the trial.

## 3 RESULTS AND DISCUSSION

### 3.1 Heat‐induced structural changes

#### 3.1.1 Viscosity and flow behavior

**FIGURE 3:**
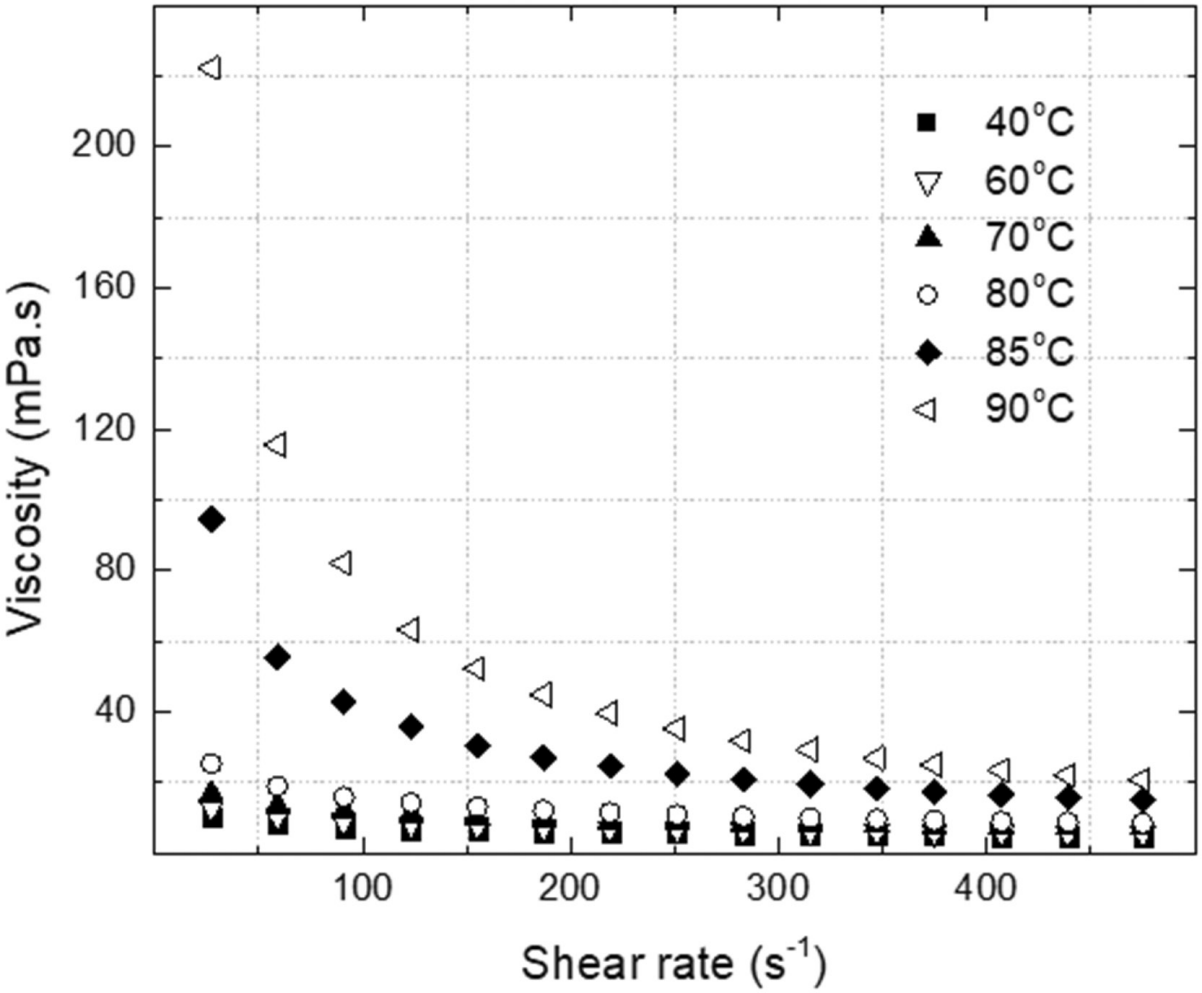
Flow behavior curves of CCE at 40–90°C temperatures

Figure 3 shows the flow behavior of CCE at (40–90°C) range temperature. The analyzed samples experienced shear‐thinning behavior over the selected temperatures, and such behavior decreased with increased shearing. It was found that coconut milk exhibited mildly pseudoplastic behavior at relatively moderate temperatures of 10–50°C due to its high‐fat content (Maghazechi et al., 2021). Moreover, the works of Simuang et al. obtained similar results at high temperatures of 70–90°C and fat content of around 15%–30%, where the coconut milk samples expressed pseudoplastic behavior (Simuang et al., 2004).

The power law model has been used for fitting the viscosity results as it was found as a better fit for CCE samples (Maghazechi et al., 2021). The viscosity buildup pattern due to the heat‐induced changes was identified by adapting the viscosity plot with the fitting model parameters *K* and *n*, since *K* is an index of viscosity and *n* values are flow index. Table 1 shows the list of the fitting model parameters *K* and *n*, which shows a clear *K* trend with increased temperature and an overall decrease in *n* values with increasing temperature.

**TABLE 1:** Power law model parameters at different temperatures

Temperature (°C)	*K* (mPa s^n^)	*n*	*R* ^2^
40	27.79 ± 0.01^f^	0.81 ± 0.01	1.000
60	34.31 ± 0.02^e^	0.75 ± 0.05	0.999
70	89.21 ± 0.3^c^	0.58 ± 0.02	0.999
80	320.60 ± 0.34^a^	0.41 ± 0.03	0.999
85	829.65 ± 0.33^b^	0.35 ± 0.04	0.999
90	1500.00 ± 0.23^d^	0.29 ± 0.02	0.999

*Note*: Values are expressed in mean ± standard deviation (*n* = 3). Means within the same column followed by different superscript lowercase letters indicate significant differences at *p* < .05 by Tukey's multiple comparisons test.

An initial increase in the viscosity was pronounced around 70°C during the heating process of CCE. This is explained by irreversible protein denaturation at high temperatures, which causes aggregation and viscosity increases (de Souza et al., 2015), followed by fat coalescences and agglomeration. The droplet interaction with increasing temperature eventually led to CCE flocculation at higher temperatures around 80°C, which led to higher viscosity (McClements, 2016). On the other hand, at 85–90°C, the *K* values rapidly increased; as a mark of viscosity divergence, this can be linked to the intermolecular association, aggregation of the unfolded protein molecules, and may bind the aggregated fat droplets together in a network (Sliwinski et al., 2003). These changes may be marked as emulsion gelation of CCE, the so‐called self‐standing gel emulsion.

Tangsuphoom and Coupland (2005) mentioned that protein–protein hydrophobic attractions following denaturation could increase the effective particle size that enhances thermal‐induced structural flocculation and eventually increases the apparent viscosity of CCE. The increase in CCE viscosity with increasing in temperature, led to consider it as an essential factor that affect the CCE fouling buildup. This was in agreement with the findings of Pichitvittayakarn et al. (2006), who concluded that the low‐flow‐rate operation resulted in a higher rate of fouling. The determined viscosity behavior of CCE was confirmed by the dynamic temperature sweep results and visual images in the following sections.

#### 3.1.2 Dynamic temperature sweep

**FIGURE 4:**
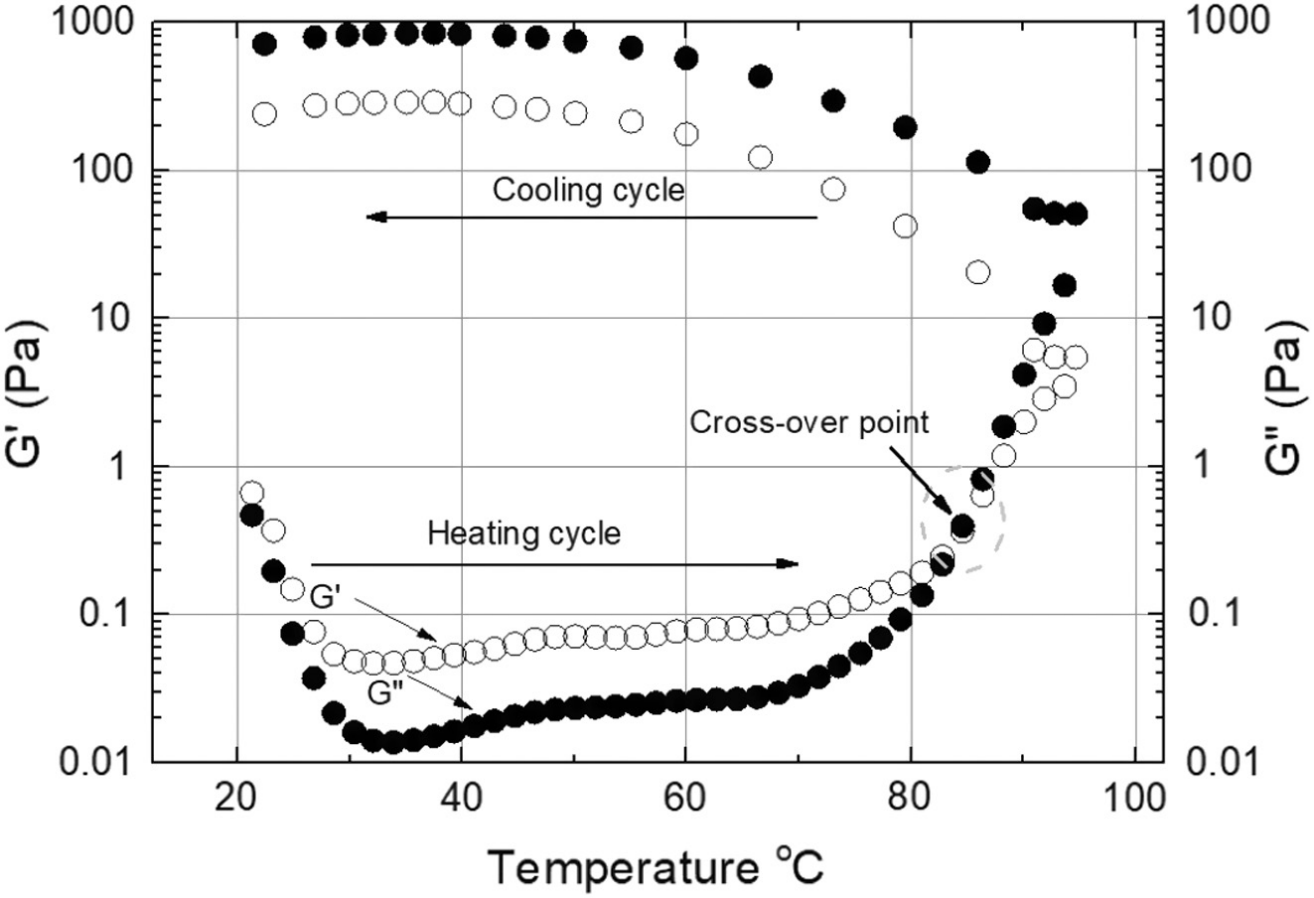
Dynamic temperature ramp curve of CCE during the heating cycle from 20 to 95°C and cooling cycle from 95 to 20°C. Open circle (○) = G″, filled circle (●) = G′.

The heat‐treatment effect on the viscoelastic property of CCE was determined using two parameters, namely the storage modulus G′, which is a measure of its elastic quantity, and the loss modulus G″, which is a measure of its viscous quantity (Lee et al., 2008). The knowledge of these moduli can be used to understand better the thermal process effect on CCE rheological properties, which eventually may lead to fouling and deposit generation. During initial heating from 20 to 45°C, there was a slight decrease in both moduli G′ and G″. More likely, these results from the melting of fat, where from 50 to 70°C, the slight increase in G″ is more likely attributed to the coalescence of the CCE (Figure 4).

Two critical regions were observed during the heating cycle, which started from 70 to 95°C. The first region was between the temperature range 70–80°C, in which the G′ and G″ values increased sharply. More likely, with increasing temperature, more proteins in the CCE denatured to a more open and less structured state. As a result, more emulsion droplets aggregated and associated with heat‐induced flocculation and aggregation of the fat droplet. Moreover, from the initial temperature of the heating cycle to 85°C, CCE exhibited a viscoelastic structure with dominant viscose liquid‐like behavior (G″ > G′). The second region was found at temperature above 85°C, with an obvious crossover of G″ and G′ that shows a gelation of flocculated CCE. The flocculated CCE and intermuscular association of the denatured proteins more likely lead to forming a three‐dimensional network of aggregated droplets that extend throughout the emulsion (Dickinson, 2013). This type of system is referred to as a particle gel. The rheological changes due to high‐temperature exposure may produce different physical properties of foulant. The 85°C may be considered a gel point, as pointed out by Taktak et al. (2021).

#### 3.1.3 Dynamic time sweep

**FIGURE 5:**
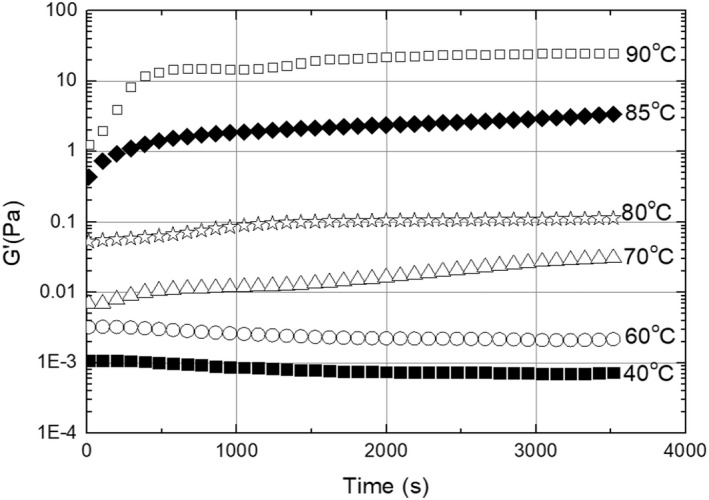
Dynamic time sweep of CCE at 40–90°C temperatures.

The dynamic time sweep test was adopted to detect the development of the CCE's structural changes in the term of storage module G′ at different temperatures ranging 40–90°C. Figure 5 shows the evolution of G′ at different temperatures. The absolute value of G′ was less significant at 40 and 60°C. At 70 and 80°C, the G′ value gradually increased, associated with protein denaturation, and aggregation (Strzelczak et al., 2021) may lead to flocculation. The G′ value then dramatically increased at 85 and 90°C to confirm the gelation of CCE due to high temperature, as discussed in the previous section. Flocculation occurs when the droplets stick to each other and form aggregates due to the minimal presence of interaction energy. The aggregates could be compact and form an expanded gel‐like structure. However, the individual droplets remain separated by a layer of thin liquid (Petsev, 2004).

#### 3.1.4 Dynamic frequency sweep

**FIGURE 6:**
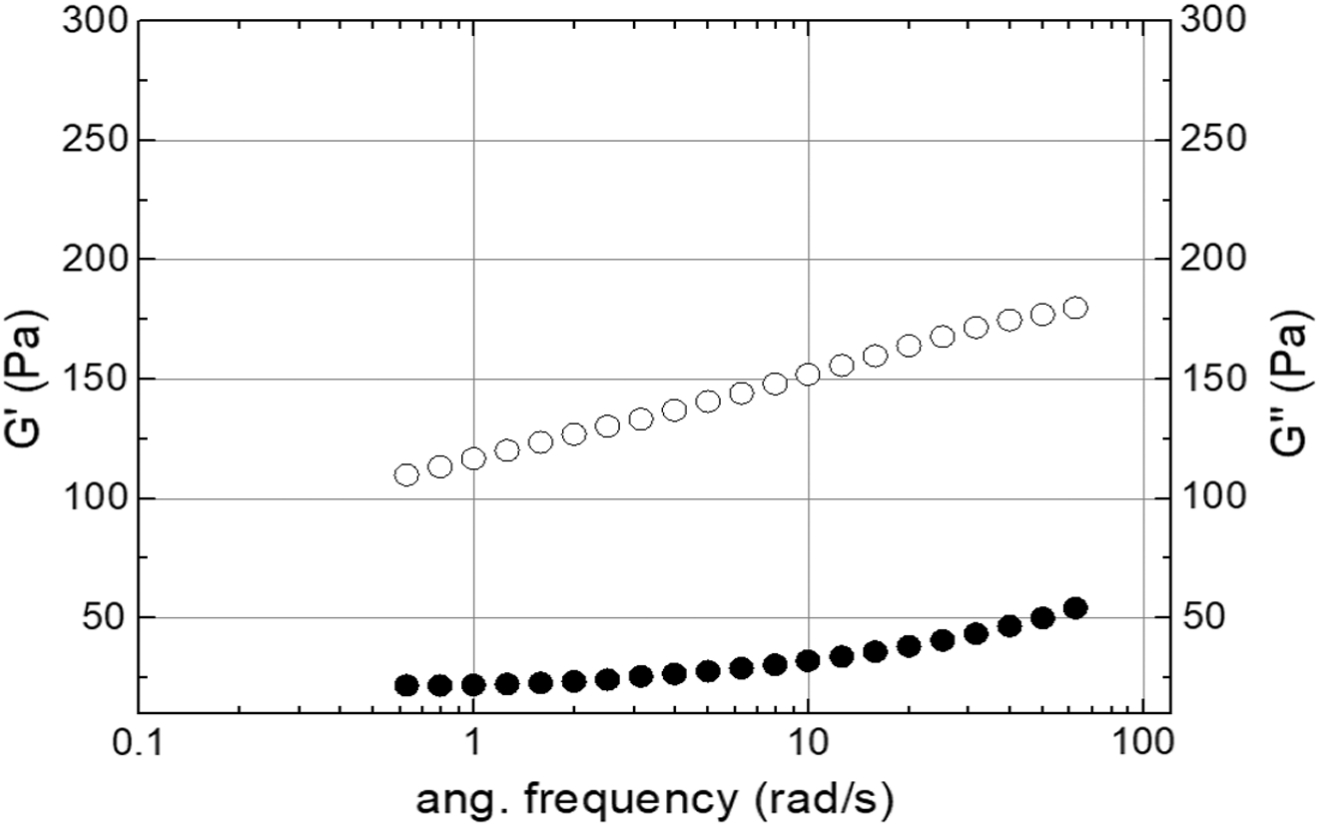
Dynamic frequency sweep average curve of CCE gel produced after heat treatment at 90°C/120 min. Open cycle = G′; filled cycle = G″. Distributions are representative of triplicates.

The dynamic frequency sweep was conducted to confirm the heat‐induced gel produced from CCE after heat treatment at 90°C/2 h (Figure 6). The plot reveals gelling properties in the heated CCE. Such a result demonstrates that after high‐temperature treatment, the CCE exhibited different behavior than at lower‐temperature treatments, consequently yielding different values of G′ and G″ values. The present test shows a higher elastic modulus G′ than viscous modulus G″ for CCE gel over the investigated frequency range, and this reflects the fact that CCE at 90°C is considered a viscoelastic system (Paseephol et al., 2008), as well as a heat‐induced gel. Mechanical spectra help classify a gel system (Alam et al., 2020). The spectra show that the ratio G′/G″ magnitude peaks were around 150 Pa. Therefore, it clarifies that CCE gel is a soft gel. Such a network cannot form a stable gel because spaces are filled with fat globules. However, the flocculated emulsion at high disperse phase volume fractions or high protein concentration in the continuous phase may form a three‐dimensional network of aggregated droplets extending throughout the emulsion (Dickinson, 2013).

#### 3.1.5 Visual assessment and measurements

**FIGURE 7:**
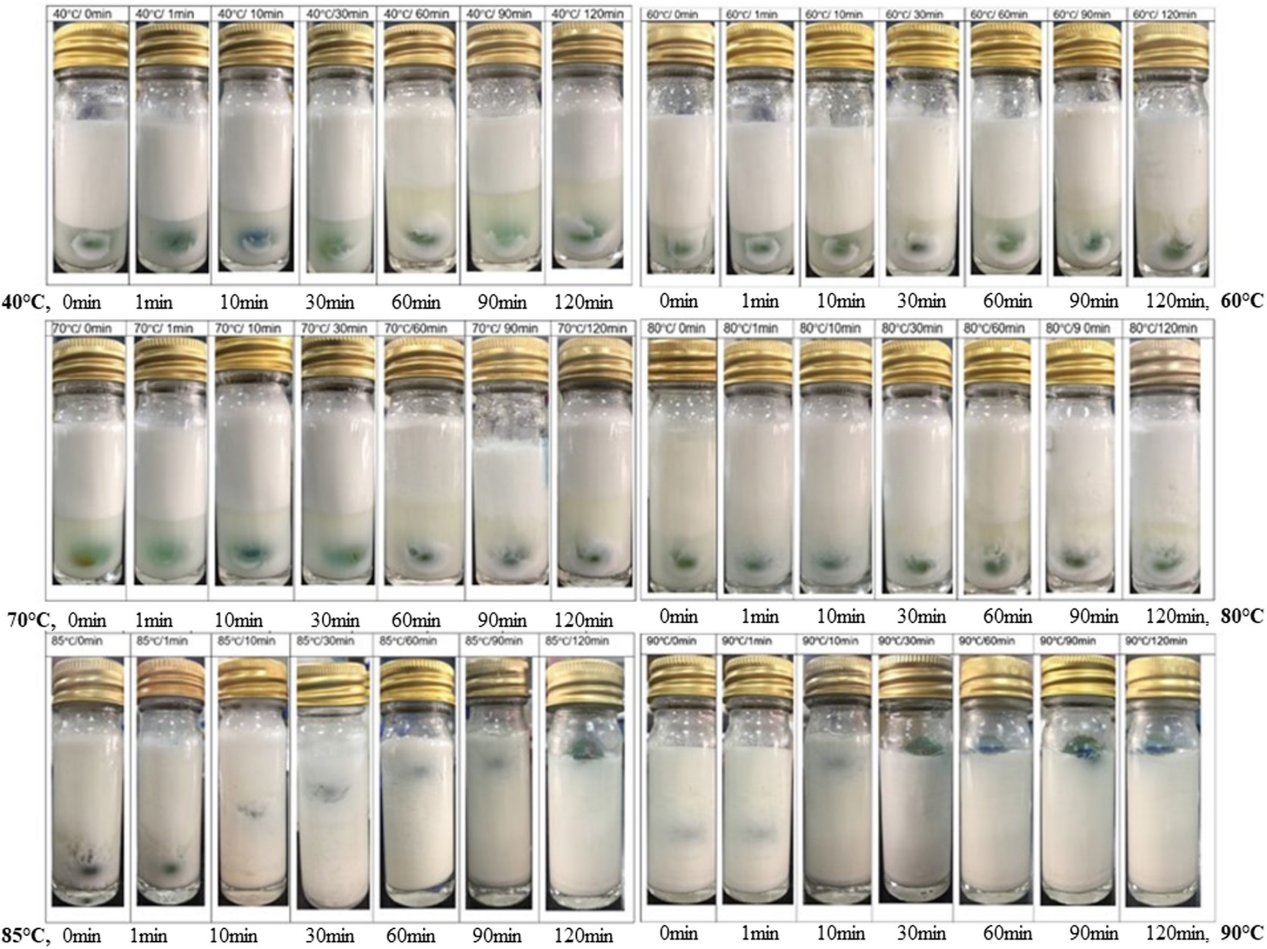
Visual assessment images of CCE heat‐induced structural changes

This assessment visually revealed dramatic structural changes in CCE during the heating process at 40, 60, 70, 80, 85, and 90°C, over the holding times of 0, 1, 30, 60, 90, and 120 min. It is apparent from Figure 7 that in the early heat treatments of 40°C till 80°C, the CCE was divided into two layers: the serum layer and the cream layer. The serum layer becomes darker (more turbid) with increased temperature over longer holding times. Such results may be due to early conformational transition, which starts from the unfolding of the heat‐liable proteins, as mentioned by Law et al. (2009). Subsequently, the denatured proteins were released from the dispersed phase to the continuous phase. This, in turn, led to increasing CCE instability. Such phase separation increases with temperature rises.

**FIGURE 8:**
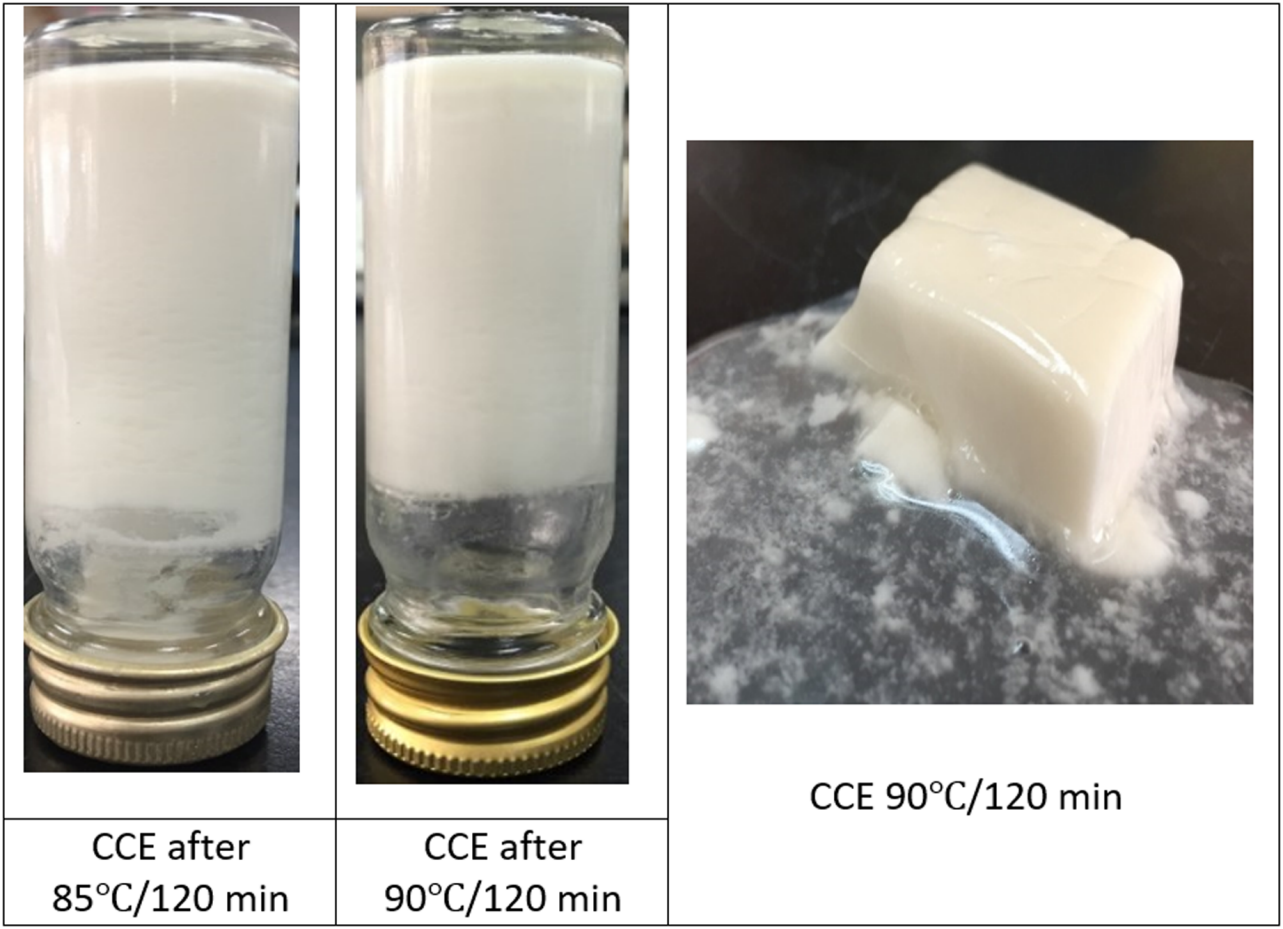
Visual images of CCE gel at 85°C/2 h and 90°C/2 h

The denatured proteins started to aggregate and concentrate in the aqueous phase in CCE by increasing the heating temperature and holding time. The layers image was modified at 85°C and 30 min of holding time and above, as well as at the treatment of 90°C total holding times, different layers appeared. The CCE is totally converting into a thick serum layer with large white aggregates dispersed in the serum. This may indicate the complete destabilization of emulsion, denaturation, and aggregation. The observed heat‐induced changes likely resulted from the CCE's denaturation and subsequent aggregation (Tangsuphoom & Coupland, 2009). At these higher temperatures of 85–90°C, higher levels of aggregated proteins were found, which correlated with the agglomeration of coconut fat. The combined effect of protein aggregates and fat agglomerate leads to CCE structural change. At temperatures ≥85°C, the concentration of aggregated protein increased, which evolved into some type of three‐dimensional, nonpourable self‐stand gel (Figure 8). Such results agree with Tangsuphoom and Coupland (2005) and McClements (2016). It is well known that heating globular proteins in aqueous solutions induce aggregation, and if the concentration is sufficiently high, this may lead to gel formation (Nicolai, 2019).

### 3.2 GFDs analysis

A preinvestigation was conducted over the temperature range 40–90°C/2 h. No GFDs were observed during the heat treatments at 40 and 60°C. The fouling deposit issue in CCE appeared at 70°C treatments and above. Based on this, CCE fouling deposits at 70, 85, and 90°C/2 h heat treatments were further investigated using microstructure analysis, FTIR peaks, and SEM plus macroimaging.

#### 3.2.1 Chemical composition

The chemical composition of protein, fat, carbohydrate, ash, and mineral of GFDs following heat treatment for 120 min at 70, 80, 85, and 90°C was calculated from the total solid content in Table 2. The GFDs showed significant variances in every component in terms of chemical composition. More protein, carbohydrates, and minerals are deposited on the surface when the temperature increases. The protein content was increased significantly (*p* < .05) from 9.43% for the GFDs at 70°C to 11.07% for the GFD at 90°C, more likely, this means more quantitively and qualitatively protein deposited with increasing temperature. Unlike dairy milk fouling, the total carbohydrate content is significant in all GFDs, as it was mentioned by Bansal and Chen (2006) that carbohydrates are not significant in dairy milk fouling. The increase in the carbohydrate and ash contents is in agreement with Saikhwan et al. (2016), that is, the carbohydrate and ash contents in the GFDs increased significantly (*p* < .05) with temperature increasing. The minimum amount of carbohydrates and ash in the GFD at 70°C were 3.10% and 1.90%, respectively, whereas the maximum amount of carbohydrates was 7.03%, and the maximum amount of ash was 4.14% in the GFD at 90°C.

**TABLE 2:** The composition of GFDs at 70, 80, 85, and 90°C/2 h

Composition (%)	Fouling deposit			
	At 70°C/2 h	At 80°C/2 h	At 85°C/2 h	At 90°C/2 h
*Proximate composition (g/100 g TS)*				
Protein	9.43 ± 0.25^a^	10.27 ± 0.05^d^	10.75 ± 0.10^c^	11.07 ± 0.10^b^
Fat	85.57 ± 0.25^d^	82.47 ± 0.42^b^	79.24 ± 0.58^a^	77.76 ± 0.28^c^
Carbohydrate	3.10 ± 0.03^c^	4.97 ± 0.2^a^	6.31 ± 0.06^b^	7.03 ± 0.01^d^
Ash	1.90 ± 0.03^d^	2.29 ± 0.03^c^	3.70 ± 0.08^b^	4.14 ± 0.15^a^
Minerals (mg/100 g)				
Iron	40 ± 0.00^b^	30 ± 0.00^c^	30 ± 0.00^d^	50 ± 0.00^a^
Phosphorus	90 ± 0.00^c^	210 ± 0.00^b^	310 ± 0.00^a^	310 ± 0.00^a^
Calcium	50 ± 0.00^a^	30 ± 0.00^b^	30 ± 0.00^b^	30 ± 0.00^b^
Sodium	90 ± 0.00^b^	100 ± 0.00^a^	100 ± 0.00^a^	100 ± 0.00^a^
Magnesium	50 ± 0.00^b^	50 ± 0.00^b^	60 ± 0.00^a^	50 ± 0.00^b^
Potassium	210 ± 0.00^b^	210 ± 0.00^b^	250 ± 0.04^a^	130 ± 0.00^c^
Chloride	170 ± 0.00^b^	170 ± 0.00^b^	130 ± 0.08^c^	200 ± 0.00^a^
Manganese	60 ± 0.00^a^	50 ± 0.00^b^	30 ± 0.00^c^	50 ± 0.00^b^

*Note*: Data are expressed as arithmetic mean (*n* = 3) ± standard deviation (2*σ)*.

Despite the highest fat content among all the GFDs, it reduced significantly (*p* < .05) with increasing the heating temperature, which is scored 85.57%, 82.47%, 79.24%, and 77.76% for GFDs at 70, 80, 85, and 90°C, respectively. This is in agreement with Pichitvittayakarn et al. (2006); in their study on the coconut cream emulsion, it was mentioned that more denaturation of proteins resulted in less ability at high‐temperature conditions to entrap fat globules onto the heating surface.

The mineral analysis of the deposit layers showed the presence of phosphorus as the main mineral, with a buildup trend. The amounts of calcium and sodium were almost the same and unchangeable with temperature differences. While the other minerals fluctuated in values, that is, temperature independent, it is unclear if minerals in the GFD layer play a key role in forming the GFD layer or are simply embedded in the deposited protein layer. There is no clear evidence in the coconut cream literature that relates mineral formation in fouling deposition. Meanwhile, Visser and Jeurnink (1997) found that protein denaturation and aggregation, with the deposition of calcium phosphate minerals, are two separate processes with different kinetics.

It may be concluded that the tendency for fat to deposit diminishes as the temperature rises, whereas the likelihood for protein, carbohydrates, and ash to deposit increases. Even though fat is the main component of the GFDs, the GFDs formation was contributed mainly by protein and, to a lesser extent, carbohydrates and ash.

#### 3.2.2 FTIR

**FIGURE 9:**
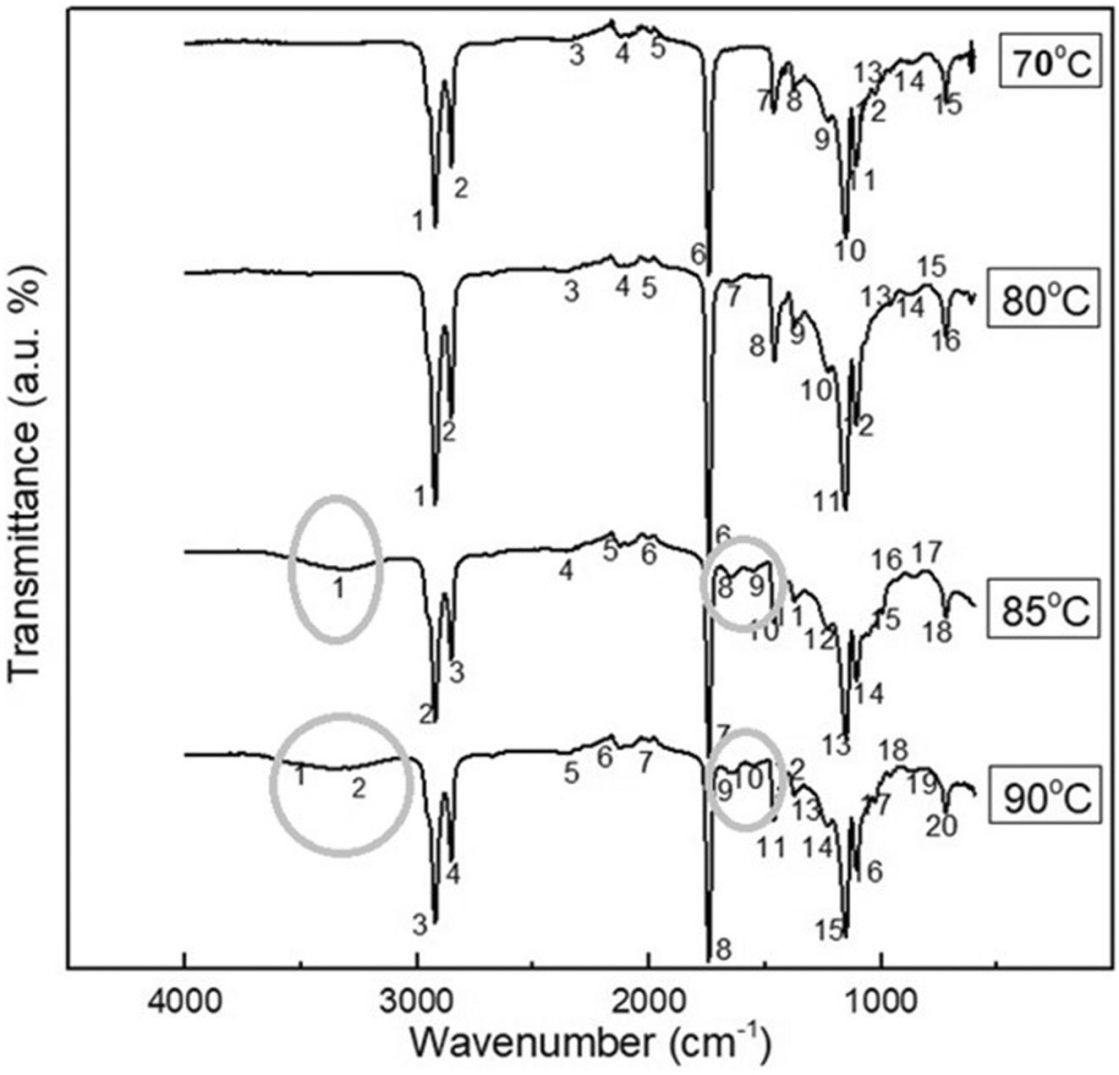
FTIR spectra of GFDs after heat treatments of 70, 80, 85, and 90°C/2 h

Fourier transform infrared (FTIR) spectra were determined for CCE fouling compositions, which were generated after the heat treatments at 70, 80, 85, and 90°C/2 h. All four deposits show the same major components, except for some new peaks that only appeared in the GFDs of 85 and 90°C/2 h. Therefore, all CCE fouling samples show peaks associated with protein, fat, and carbohydrates (Figure 9).

The characteristic peaks around (2923, 2855, and 1744 cm^−1^) can be assigned to the lipids (Mizutani et al., 2004). While the peaks around 1229, 1027, 967, and 884 cm^−1^ were attributed mainly to carbohydrates (Barth, 2007), the peaks around 1154, 1110, and 700 cm^−1^ could be identified as proteins (Saikhwan et al., 2016). However, peaks around 700 cm^−1^ were also observed in coconut oil (Lu & Tan, 2009). Interestingly, four novel peaks appeared in the 90°C/2 h deposit results, and 3 of the new peaks also appeared in the 85°C/2 h deposit. Two of them were at 3526 and 3295 cm^−1^, which were attributed to water and amide A band protein, respectively (Ye et al., 2017), while the other two peaks at 1644 and 1549 cm^−1^ were from amide I and amide II proteins, respectively (Carbonaro et al., 2008). Such specific new appeared peaks were somehow explained differently in other research works. For example, it was mentioned by Wang et al. that the peaks around 1540 cm^−1^ are an indicator of the combined proteins (Wang et al., 2018). More likely, this is attributed to some types of proteins with high aggregation temperatures. Also, Ball and Jones reported that the change around the peak 1632 cm^−1^, and the shoulder around 1650 cm^−1^ that start to rise are consistent with those occurring on heat gelation of proteins (Ball & Jones, 1995). Due to the novel peaks identified in conjunction with the formation of a shoulder in the GFDs at 85 and 90°C, it is possible to conclude that the fouling deposits begin to transform to a heat‐induced gel approximately at temperature ≥85°C. Most likely, this is related to the construction of the GFD gel, which was caused by the deposition of more proteins and the production of new aggregates. This finding may be supported by the results of a previous chemical analysis of GFDs and the results of a subsequent SEM investigation.

#### 3.2.3 SEM and macroimaging

**FIGURE 10:**
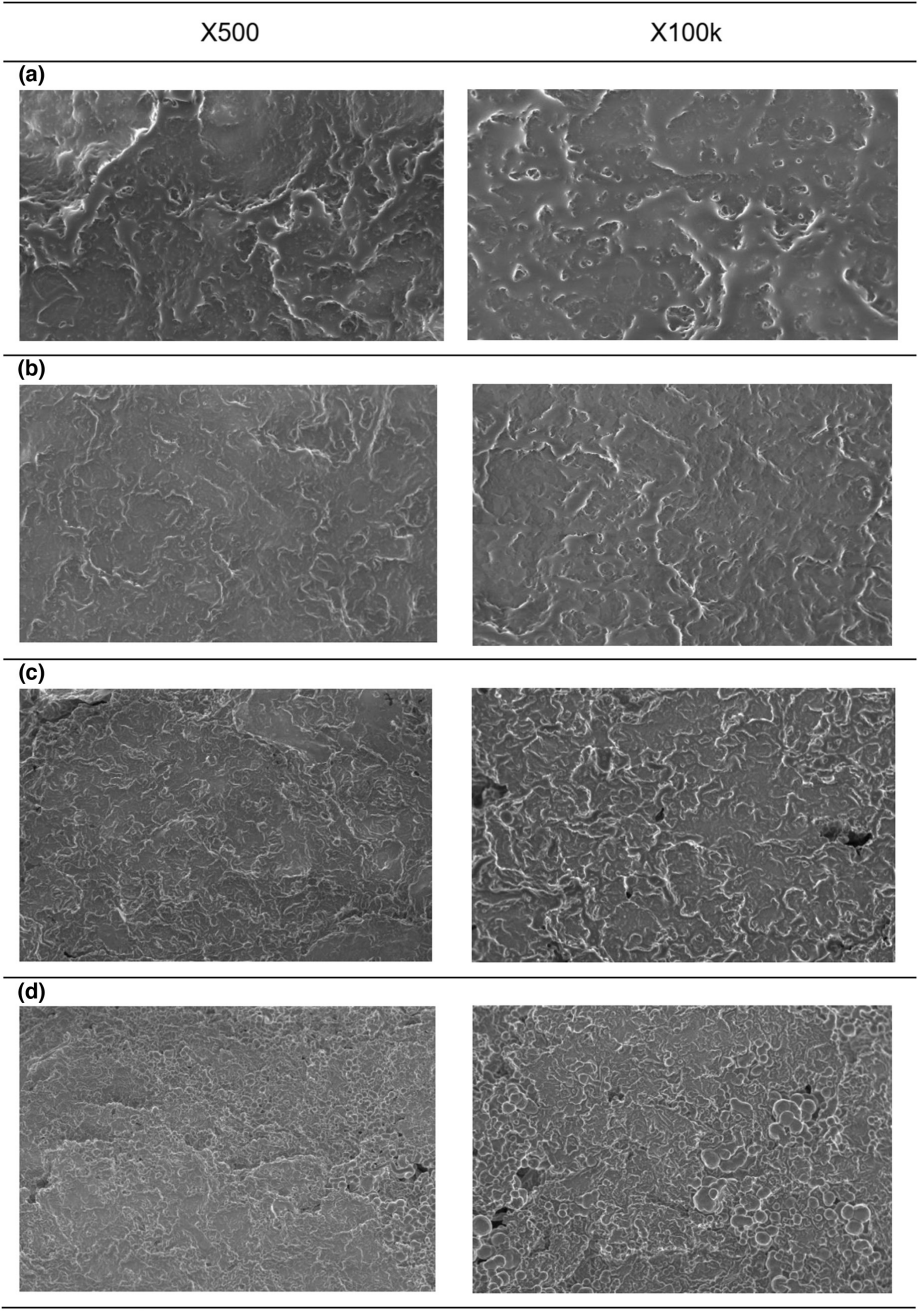
SEM images of GFDs after heat treatment of (a) 70°C, (b) 80°C, (c) 85°C, and (d) 90°C/120 min

The morphology and topography of GFD were qualitatively analyzed using SEM. Figure 10 depicts four different morphologies of foulants due to heat treatments at 70, 80, 85, and 90°C/120 min.

The GFDs' morphological images alter substantially as the temperature goes up. At 70°C and 80°C, the GFD was a homogeneous layer of deposit and the presence of a thin continuous protein matrix (Figure 10a,b). Furthermore, there were no porous deposit structures, and the deposit structures were compact. Meanwhile, the deposit morphology began to change after the GFD at 80°C, and this shift was more noticeable with the deposit at 90°C. The GFDs at 85 and 90°C began as a multilayer continuous phase with embedded particles between the layers.

In addition, more irregular pores form, which are more likely filled with water; the coconut milk deposit is full of water, according to Law et al. (2009). The same reference mentioned that coconut milk fouling deposits are a semisolid material. As a result, a new network of deposit morphologies has developed. Because of the aggregated protein and fat network, the GFD at 90°C formed a dense and gel‐like layer. This conclusion agreed with Pichitvittayakarn et al. (2006) findings.

**FIGURE 11:**
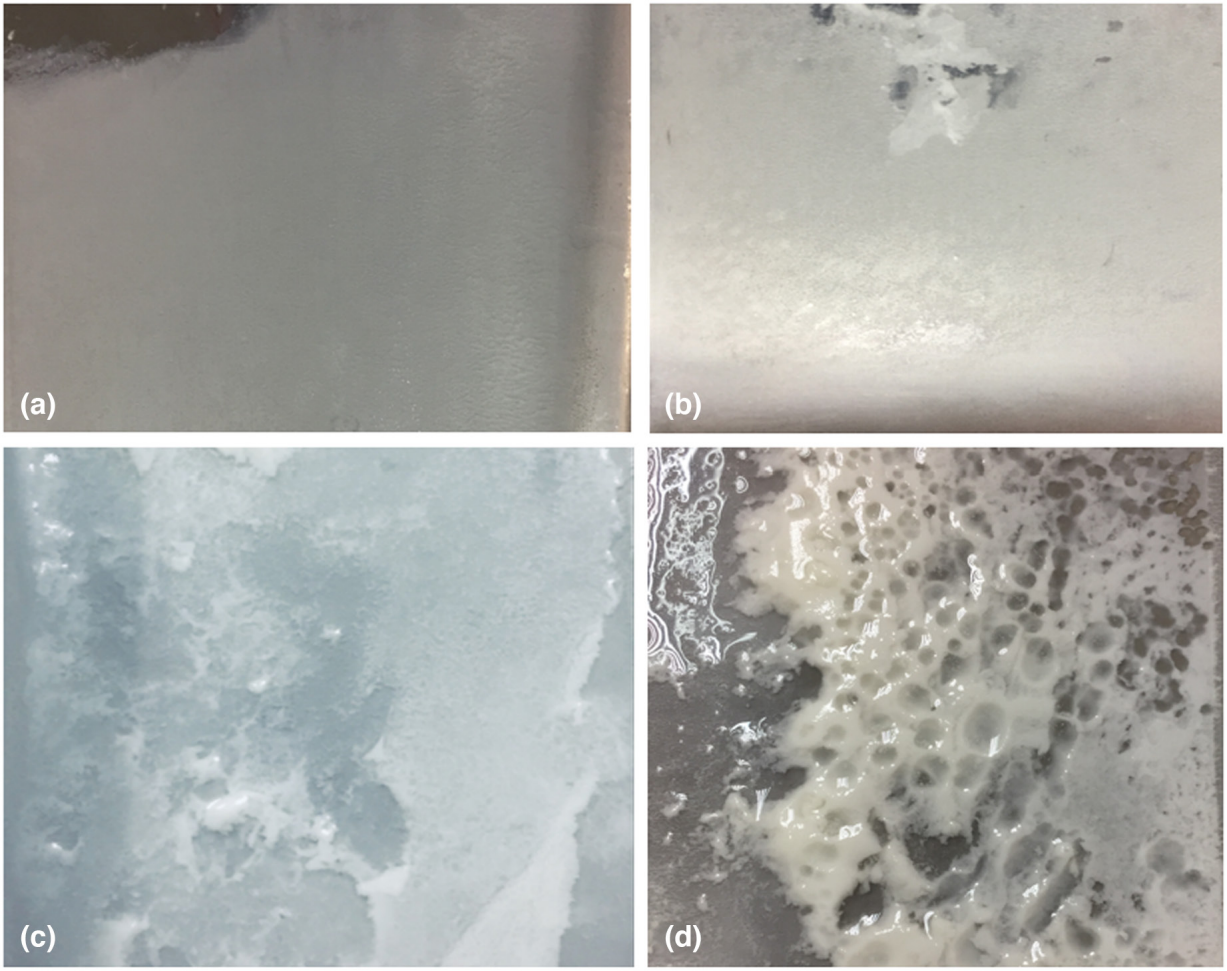
Macroimages of GFDs after the heat treatments of (a) 70°C, (b) 80°C, (c) 85°C, and (d) 90°C/120 min

The macroimages for the GFDs are shown in Figure 11. The GFDs were greasy and covered in agglomerated oil at all heating temperatures. The GFD developed as a thin‐film‐like deposit comprising small protein aggregates between 70 and 80°C (Figure 11a,b). The continuous protein matrix was more visible in the macroimages of the GFDs at 85 and 90°C (Figure 11c,d), which showed multiple layers. The GFD at 90°C was thicker and appeared as a gel‐like layer than the GFD at 85°C. The chemical composition, FTAR, and SEM confirmed that the GFD at 90°C started to produce some gel layer particles filled with fat clusters.

## 4 CONCLUSIONS

The rheological analysis of heat‐induced structural changes in the CCE bulk during heating at 60–90°C demonstrated a considerable increase in viscosity and G′ during the experiment. The results showed that CCE bulk had experienced significant structural changes, beginning with instability, protein denaturation, aggregation, and fat cluster embedding in the protein matrix and progressing to gel particle production. The increase in the CCE bulk viscosity may significantly increase GFDs in the manufacturing process. The GFDs were investigated over a heating treatment 40–90°C. The GFD began to develop on the heating surface at temperatures around 70°C. When the heating temperature increased from 70 to 90°C, more components in the CCE bulk lost their rheological properties and were collected as a GFD layer on the heating surface. Moreover, novel FTIR peaks for GFDs around temperature ≥85°C were observed. In such temperatures, the GFDs emerged as a gel‐like GFD layer. It is worth noting that GFDs in the CCE are caused mainly by protein denaturation, aggregation, gelation, and, to a lesser extent, carbohydrates and ash. Understanding the GFDs process and seeking to alleviate the GFDs issue require considering heat‐induced structural changes in the CCE bulk. A study on the deposition of CCE fouling is urgently required.

## CONFLICT OF INTEREST

The authors declare no conflict of interest.

## FUNDING INFORMATION

This study was supported by the Higher Education Ministry of Malaysia, Fundamental Research Grant Scheme [203.PTEKIND.6711812, 2019]

## ETHICS STATEMENT

This study does not involve any human or animal testing.

## Data Availability

The data that support the findings of this study are available from the corresponding author upon reasonable request.
